# Editorial: Atrial Fibrillation: Technology for Diagnosis, Monitoring, and Treatment

**DOI:** 10.3389/fphys.2022.848096

**Published:** 2022-02-23

**Authors:** Omer Berenfeld, Valentina D. A. Corino, Axel Loewe, Juan Pablo Martínez, Jose F. Rodriguez Matas

**Affiliations:** ^1^Center for Arrhythmia Research, University of Michigan, Ann Arbor, MI, United States; ^2^Biosignals, Bioimaging and Bioinformatics Laboratory (B3Lab), Department of Electronics, Information and Bioengineering (DEIB), Politecnico di Milano, Milano, Italy; ^3^Institute of Biomedical Engineering, Karlsruhe Institute of Technology (KIT), Karlsruhe, Germany; ^4^Biomedical Signal Interpretation and Computational Simulation Group, Aragón Institute of Engineering Research, IIS Aragón, Universidad de Zaragoza, Zaragoza, Spain; ^5^Centro de Investigación Biomédica en Red en Bioingeniería, Biomateriales y Nanomedicina, Zaragoza, Spain; ^6^Laboratory of Biological Structure Mechanics (LaBS), Department of Chemistry, Material and Chemical Engineering “Giulio Natta”, Politecnico di Milano, Milano, Italy

**Keywords:** atrial fibrillation, atrial disease, AF detection, ablation, mapping, machine learning, atrial remodeling

## Introduction

Atrial fibrillation (AF) is the most common sustained clinical arrhythmia. With a 2.4-fold risk increase, AF is the leading cause of embolic stroke. It also increases the risks for heart failure 5-fold and mortality 2-fold (Benjamin et al., 2017; Roth et al., 2017). AF is reaching an epidemic proportion estimated to affect 0.51% of the population and its prevalence is estimated at more than 37 million worldwide. That prevalence increased by a 33% in the last 20 years and is expected to increase by more than 60% in 2050 (Chugh et al., 2014; Lippi et al., 2021). Overall, AF is a major societal burden with immense financial costs associated with the care of patients, mostly on hospitalization and complications (Kowalski et al., 2022). It is estimated that the total annual incremental costs of AF care can reach $26 billion in the US (Kim et al., 2011) and AF costs across several European countries account for 0.28–2.60% of their total healthcare spending (Chugh et al., 2014; Zoni Berisso et al., 2017; Velleca et al., 2019).

The reasons for the high burden of AF may lay in its heterogenous, multi-factorial and progressive nature; despite intense research efforts, its initiation, sustenance and termination mechanisms are still poorly understood, and therapy remains suboptimal (Heijman et al., 2018). It is widely accepted that both AF research, healthcare delivery and outcomes can be improved by advancement of technology (Kowalski et al., 2022). This Research Topic presents a collection of 37 original and review papers focusing on technological challenges and advances for improved understanding of AF and better diagnosis, monitoring and management of the arrhythmia. A total of 262 students and faculty of diverse scientific background have contributed to the papers and highlight the important role of a multi-disciplinary research, particularly by next generation investigators, in advancing AF therapy.

## AF Detection and Stratification

Early diagnosis of AF, most commonly achieved through analysis of ECG signals, could help in timely therapy and machine learning (ML) approaches have been proposed for improvement of the ECG analysis for AF detection. In this issue Rouhi et al. utilize the ML game theory Shapley Additive exPlanations technique along with random forests (RF) decision scheme to rank the importance of the ECG features to enhance classification of ECG signals and AF detection. Garcia Isla et al. use heart rate variability and ECG morphological features together with a RF algorithm to classify premature atrial complexes. Halvaei et al. propose a convolutional neural network (CNN) for transient noise identification in AF detection showing that a reduction of false AF detections can be significantly reduced by identification of transient noise. Today, identification of AF episodes in long-term ECG recordings is mostly performed manually. To reduce the workload for reviewers, the ECG time-series can be reconfigured to present sequential beats in a stacked form termed electrocardiomatrix (ECM) to assist in visualization of long-term ECG information. Salinas et al. developed a CNN approach based on ECM images for automatic detection of brief AF episodes.

Apart from effective early AF detection, adequate patient stratification is important for improving the success rate of first ablation. In the work by Saiz-Vivo et al., clinical and heart rate variability features extracted from an implantable cardiac monitor were used to predict rhythm output using an ensemble classifier formed as the weighted combination of three classifiers: Support Vector Machine, Classification and Regression Trees, and K-Nearest Neighbor. The proposed method aims for a more effective pre-ablation patient selection. McCann et al. propose ECG measures of organization that can be used to identify patients unlikely to benefit from catheter ablation, albeit without association with ablation outcome.

## AF Characterization and Ablation

When pharmacological cardioversion fails, atrial arrhythmias are typically treated with catheter ablation. In such case, characterizing the atrial electrical activity and structure in patients may constitute an important step toward a successful intervention. This step mostly relies on various methods for electrical activity and tissue characteristics mapping to identify the arrhythmogenic substrate. Salinet et al. review the use of non-invasive electrocardiographic imaging for AF characterization for ablation guidance and discuss the technological and validation requirements. Muffoletto et al. propose image-based computational modeling combined with deep learning to assist in catheter ablation strategy planning. Nothstein et al. present an openly available pipeline for the automatic evaluation of atrial signals recorded during pacing from other electrodes of the same circular catheter even with low signal-to-noise ratio. Williams et al. developed the open-source OpenEP software and database structure for the analysis of electroanatomic mapping data from a number of commercial systems. A network theory approach is used in Vila et al. to identify the electric propagation patterns in atrial flutter allowing for the automatic identification of reentry circuits facilitating localization of ablation targets in patients. The dominant frequency (DF) of atrial electrograms during AF reflects local activation rate, with highest DF sites potentially driving AF. Li et al. developed a tool to automatically detect recurrent spatial DF patterns in persistent AF patients. Their method successfully identifies and quantifies the spatiotemporal repetition of high DF sites showing that recurring patterns offer a more comprehensive dynamic insight of persistent AF. The potential benefit of ablating such regions remains to be shown. An important element of catheter ablation intervention is a pacing protocol to test for non-inducibility of arrhythmia post-ablation. Azzolin et al. perform an *in-silico* investigation searching for a standardized protocol to induce arrhythmia after an intervention. They propose a novel method of pacing at the end of the effective refractory period for assessment of arrhythmia vulnerability. The openly available protocol can become a standard for *in silico* and clinical arrhythmia inducibility testing.

Successful AF termination by ablation is thought to dependent on the detection of drivers and their interactions with the atrial activity at large. Among the different possible driver types, reentrant and rotor patterns have gained the most attention. Spector et al. show in a computational propagation model that the degree to which a focal reentrant driver and the surrounding chaotic activation interact depends on the relative characteristics of the anatomical and functional (rotor) reentrant substrates. Ganesan et al. developed a quantitative birth-death framework providing insight into the wavelet and rotor dynamics in AF and their spontaneous termination. In the clinic, rotor detection typically relies on signals recorded by multi-electrode catheters, which can be influenced by a number of structural parameters and therefore, an efficient design is key. Bartolucci et al. propose a tool for testing the ability of different catheter shapes to detect rotors in different conditions that could assist in the design of new mapping catheters.

## Risks for and From AF

A possible factor contributing to AF is atrial stretch. Lee et al. show that higher left atrium (LA) wall stress was associated with poorer rhythm outcomes after catheter ablation. Further, Eichenlaub et al. report in a fifty-patient study how LA hypertension, electrical conduction slowing, and mechanical dysfunction are all associated with relevant atrial cardiomyopathy. Their study suggests the use of these atrial cardiomyopathy markers for risk stratification of arrhythmia recurrence following catheter ablation.

Heterogeneous intra-atrial conduction is known to facilitate both initiation and perpetuation of AF. In the work by Gaeta et al., a method for high resolution measurements of local activation time (LAT) differences for characterizing local changes in myocardial conduction velocity was developed. The methodology was tested with *in vivo* bipolar electrograms (EGMs) which showed a good agreement with standard LAT annotations and unipolar waveforms. Ye et al. developed a patient-specific signal fingerprint based on EGM features to characterize the severity and extensiveness of heterogeneity in conduction. Riccio et al. simulate impulse propagation to demonstrate a modified version of the omni-polar electrogram method for improved characterization of substrate and propagation, reduction of residual sensitivity to directionality over the standard approach and improved robustness against noise. The role of substrate heterogeneities causing conduction velocity inhomogeneities—often related to fibrosis—was investigated using a computational model by Pagani et al. Their simulations show how substrate characteristics may contribute to inducing and sustaining arrhythmias and demonstrate how localized reentries tend to anchor in areas of severe slow conduction in persistent AF.

The consequences of AF are not only related to heart rhythm and rate alterations but could also importantly lead to thrombus formation, most likely in the LA appendage. Sanatkhani et al. imaged the LA appendage in a cohort of 16 AF patients and simulated the hemodynamic therein. Quantification of the appendage geometrical complexity and its characteristic time of blood residency during stasis could enhance ability to stratify stroke risk in AF patients. Similarly, Paliwal et al. investigate with personalized LA hemodynamic *in silico* modeling the mechanisms for increased stroke risk in patients with atrial fibrotic remodeling, indicating that patients with high LA fibrotic burden have a higher probability for clot formation and thus a higher risk of stroke.

## AF and the Autonomous Nervous System

The autonomic nervous system (ANS) has an important role in the generation and maintenance of cardiac arrhythmias. Therefore, characterizing ANS activity during AF may facilitate personalized treatment. The activity of the ANS during AF can be quantified in a number of ways as for example through the magnitude of respiratory induced modulation of the f-wave frequency during AF as shown by Abdollahpur et al. The ANS consists of multiple ganglionated plexi (GP) and axons, which innervate the neighboring atrial myocardium and control their electrophysiological properties. GP ablation has been associated with a decreased risk of AF recurrence; however, accurate localization of GPs is required for ablation to be effective. Celotto et al. performed an *in-silico* study and propose a method to locate the GPs by a robust analysis of the small amplitude unipolar signals during the repolarization phase of action potentials. Zhang et al. report that superior left GP ablation suppressed chronic AF in a chronic obstructive sleep apnoea canine model of sympathovagal hyperactivity inhibition. From the same group, Guo et al. suggest that low level vagus nerve stimulation could decrease the inducibility of AF in that animal model.

## Electrophysiological Remodeling and Pharmacological Treatments of AF

Several articles in this Research Topic demonstrate how underlying AF factors may be inherited, remodeled, or both. Computational studies show increased propensity for AF in gain-of-function genetic mutations encoding potassium channel proteins (Belletti et al.) and metabolic hypokalemia (Clerx et al.). Big and small conductance calcium activated potassium channels (BKCa and SKCa) and two-pore-domain potassium channels (TASK1) are among the family of channels experiencing electrical remodeling during AF. The study by Jakob et al. investigates the electrophysiological phenotype of cultured fibroblasts from patients with sinus rhythm and AF and observed the presence of BKCa channels with reduced open probability in AF, confirming previous studies. The identification of channels principally expressed in the atria and remodeled in AF patients has motivated the study of atria-selective pharmacological therapies. Along this line of thought, Darkow et al. studied the mRNA expression of atrial and ventricular SKCa channels in AF and heart failure (HF) patients. Their study reports a downregulation of KCNN2 in AF patients but no significant difference between AF and HF patients, suggesting that those channels are not likely to be an atria-selective target, especially in failing human hearts. The efficacy of SKCa inhibition to terminate AF and its dependence on AF duration is further investigated in Fenner et al. on an equine model of persistent AF. The study reports divergent effects on the right and left atria that impeded cardioversion and leave an open door for further investigations. Wiedmann et al. report that pharmacological inhibition of TASK1 can be employed for rhythm control in a porcine model of persistent AF.

AF can also remodel calcium channels and affect Ca^2+^ diffusion and handling. In a computational simulation study by Vagos et al., downregulation of the L-type calcium current induced by atrial tachycardia is shown to play a predominant role in the calcium silencing. On the other hand, AF can induce overexpression and oxidation of calcium/calmodulin-dependent protein kinase II (CaMKII) that could severely affect calcium flux through the sarcolemma and intracellular handling within the cell, promoting arrhythmic behavior of atrial cells. Wang et al. performed an *in-silico* study in a mouse atrial cell model showing how CaMKII oxidation and overexpression would affect the electrophysiological behavior and arrhythmogenic delayed afterdepolarization mechanisms.

Finally, pharmacological rate control during AF can be an alternative to attempting to terminate AF in rhythm control strategies. Karlsson et al. developed a network model to estimate conduction delay and refractory period of the atrio-ventricular node from ECG data from a patient at baseline and during treatment with a heart rate control drug. They demonstrate the ability of the methodology to assess the effect of rate control drugs.

## Conclusion

We are excited to present a collection of studies describing recent laboratory, computational and clinical AF approaches and investigations. We hope that scientists, engineers and clinicians, as well as patients, interested in the Research Topic will find this overview of basic and translational research trends to be inspiring and promoting improved understanding and therapies of AF.

## Author Contributions

JR wrote the first draft of the manuscript. OB wrote sections of the manuscript and performed a general editing. All authors contributed to manuscript revision, read, and approved the submitted version.

## Funding

OB acknowledges support by the National Institutes of Health National Heart, Lung, and Blood Institute grants R01-HL118304 and R21-HL153694, as well as research grants from Abbott, Medtronic Inc. and CoreMap Inc. JM acknowledges support by Spain Ministerio de Ciencia e Innovación grant PID2019-104881RB-I00 and reference group T39-20R (Aragón Government cofounded by FEDER 2014-2020 Building Europe from Aragon). VC, AL, JM, and JR acknowledge the support of the European Union's Horizon 2020 Research and Innovation Program under the Marie Skłodowska-Curie Grant Agreement No. 766082. AL acknowledges support by the German Research Foundation (DO 637/22-3 and LO 2093/1-1), and the European Union's Horizon 2020 Research and Innovation Programme under the Marie Skłodowska-Curie Grant Agreement No. 860974 and from the European High-Performance Computing Joint Undertaking EuroHPC (JU) under Grant Agreement No. 955495. The JU receives support from the European Union's Horizon 2020 Research and Innovation Programme and France, Italy, Germany, Austria, Norway, and Switzerland.

## Conflict of Interest

OB was co-founder and Scientific Officer of Rhythm Solutions, Inc. and is a co-founder of Cor-Dx LLC. The remaining authors declare that the research was conducted in the absence of any commercial or financial relationships that could be construed as a potential conflict of interest.

## Publisher's Note

All claims expressed in this article are solely those of the authors and do not necessarily represent those of their affiliated organizations, or those of the publisher, the editors and the reviewers. Any product that may be evaluated in this article, or claim that may be made by its manufacturer, is not guaranteed or endorsed by the publisher.
